# Combination of cafeteria diet with intraperitoneally streptozotocin in rats. A type-2 diabetes model

**DOI:** 10.1590/ACB360702

**Published:** 2021-08-23

**Authors:** Mirelly Marques Romeiro Santos, Andressa Carolina Farias Pereira Subtil Cavalcante, Luane Aparecida do Amaral, Gabriel Henrique Oliveira de Souza, Bárbara Suzuki dos Santos, Luciane Candeloro Portugal, Felipe Franscisco Bittencourt, Thiago Troquez, Bruna Paola Murino Rafacho, Priscila Aiko Hiane, Elisvânia Freitas dos Santos

**Affiliations:** 1 MSc. Program in Health and Development in the Mid-western Region – Universidade Federal do Mato Grosso do Sul - Campo Grande (MS), Brazil.; 2 Nutritionist. Faculty of Pharmaceutical Sciences, Food and Nutrition - Universidade Federal do Mato Grosso do Sul - Campo Grande (MS), Brazil.; 3 Food Technologist. Faculty of Pharmaceutical Sciences, Food and Nutrition - Universidade Federal do Mato Grosso do Sul - Campo Grande (MS), Brazil.; 4 Pharmacist. Institute of Biology - Histology Laboratory - Universidade Federal do Mato Grosso do Sul - Campo Grande (MS), Brazil.; 5 PhD. Institute of Biology - Histology Laboratory - Universidade Federal do Mato Grosso do Sul - Campo Grande (MS), Brazil.; 6 PhD. Laboratory of Clinical Analysis - Universidade Federal da Grande Dourados – Dourados (MS), Brazil.; 7 PhD. Faculty of Pharmaceutical Sciences, Food and Nutrition - Universidade Federal do Mato Grosso do Sul - Campo Grande (MS), Brazil.; 8 PhD. Program in Health and Development in the Mid-western Region – Universidade Federal do Mato Grosso do Sul - Campo Grande (MS), Brazil.

**Keywords:** Liver, Hyperglycemia, Body Weight, Pancreas, Models, Animal

## Abstract

**Purpose:**

To develop a model of induction of type-2 diabetes (DM2) by combining low doses of streptozotocin (STZ) and a cafeteria diet.

**Methods:**

Forty male Wistar rats (200 g) were allocated into four groups: control (non-diabetic, n = 10); STZ 30 mg/kg (diabetic, n = 10); STZ 35 mg/kg (diabetic,n = 10); and STZ 40 mg/kg (diabetic, n = 10). DM2 was induced with a single intraperitoneal injection of STZ after four weeks of cafeteria diet in the three diabetic groups. All animals were evaluated as for anthropometric, and biochemical analyses, as well as liver, kidney and pancreas histological analyses.

**Results:**

Lower weight gain, higher water intake, higher Lee index, hyperglycemia and modified total protein, urea, alpha-amylase, as well as insulin resistance, hepatic steatosis, pancreas, and kidney injury were observed in animals treated with 35 and 40 mg/kg of STZ.

**Conclusions:**

The results show that the experimental model using cafeteria diet associated with 35 mg/kg of STZ is a low-cost model and efficient in order to develop DM2, confirmed by the presence of polydipsia, hyperglycemia, altered biochemical tests, insulin resistance and damages to the liver, pancreas and kidney, which is similar to the disease found in humans.

## Introduction

In 2016, the estimate was 422 million diabetic adults worldwide. By 2035, the number of adults with diabetes is expected to increase to 592 million^1^. The number of people with diabetes increases every year, being thetype-2 diabetes (DM2) found in more than 90% ofthe disease cases^2^.

DM2 is characterized mainly by imperfections in the action and/or secretion of insulin and the regularization of hepatic glucose production. Different organs are involved in DM2 development, including pancreas (with loss of cell mass and function, impaired insulin secretion of beta cell and dysregulated glucagon secretion by the alfa cell), liver (increased hepatic glucose output), skeletal muscle (reduced peripheral glucose uptake and insulin resistance), kidneys (increased glucose reabsorption), brain (increased appetite and lack of satiety), small intestine (increased rate of glucose absorption), and adipose tissue (reduced peripheral glucose uptake and insulin resistance)^2^.

The major factors that increase the risk of developing DM2 include unhealthy habits, such as sedentary lifestyle, smoking, overweight and obesity, increased consumption of unhealthy diets (containing high levels of red meat and processed meat, refined grains and sweetened beverages), as well as the interaction of genetic and metabolic factors^3^.

The cafeteria diet is a model used in animal experiments to imitate the Western food model, which is highly palatable and has a marked caloric value^4^^,^ 5. This diet is mainly composed of foods rich in saturated fat and refined sugar, such as: sausages, chocolates, cookies, soft drinks, snacks, condensed milk, among other, which are added to a standard diet^5^^,^ 6. This diet model has been shown to be efficient in inducing several metabolic changes: hyperinsulinemia, hyperglycemia, glucose intolerance, increased adiposity, and hepatosteatosis, as well as greater food consumption and increased weight gain in experimental animals^5^^,^ 7.

Cytotoxic drugs used to chemically induce DM in experimental animals have been widely used for studying drugs and prophylactic, metabolic, hormonal, and morphological factors during and after the induction of the diabetic state^8^. Streptozotocin (STZ) is a particularly toxic agent for beta pancreatic cells. It is widely used to induce diabetes in rats^9^. Low doses of STZ, when associated with a hypercaloric diet, have proven efficient in resembling DM2 in humans^10^. Hypercaloric diet leads to increasein adiposity and decrease in insulin efficacy, especially in peripheral tissues10. However, there is a great variation in STZ doses (25-50 mg/kg)^9^^,^ 11 ^,^ 12. The standardization of an experimental model is the starting point for several studies.

Thus, due to the wide variation among doses used in already published studies and the different environments and behavior of experimental animals, including ecological factors, the aims of the present study were to establish and standardize a DM2-induction model using three different doses of STZ associated with a cafeteria diet in Wistar rats.

## Methods

### Experimental design

The research project was approved by the Ethics Committee (Protocol no. 895/2017) of Universidade Federal de Mato Grosso do Sul (UFMS). Forty male Wistar rats (approximately 200 g) were obtained from the Central Bioterium of UFMS.

The animals were housed in polypropylene boxes (two animals per box) in controlled environmental conditions: temperature at 22 ± 2°C, relative humidity of 50-60%,light/dark cycle of 12 hours, and control of water intake. The diet was monitored twice a week during the experiment. After diabetes induction, water control was performed four times a week due to polydipsia. For calculations, the difference between the offered quantity and the remaining quantity of food was considered.

### Experimental groups and diet

**Figure 1:**
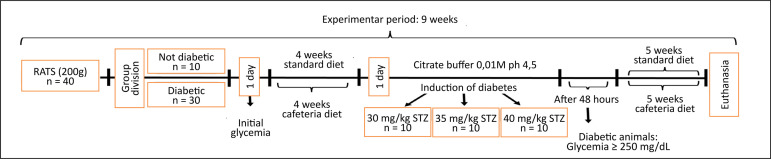
Study design.

The rats were allocated into a control group (C, n = 10)and three different diabetic groups: 30 mg/kg STZ (STZ30, n = 10), 35 mg/kg STZ (STZ35, n = 10) and 40 mg/kg STZ (SZT40, n = 10), as shown in Fig. 1. The animals in the control group were fed with commercial chow. The animals in the diabetic groups were fed with cafeteria diet containing 35% commercial chow, 10% lard, 10% peanuts, 10% chocolate powder, 10% chocolate wafer biscuit, and 25% refined sugar, based on Pérez-Echarri*et al*.^13^, for nine weeks.

The cafeteria diet had the following composition: 11% proteins, 60% carbohydrates, and 21% lipids with an energy content of 4.7 kcal/g, whereas the commercial chow (Nuvilab, Quimtia, Colombo, PR, Brazil) presented 22% proteins, 55% carbohydrates, and 5% lipids with an energy content of 3.6 kcal/g.

### Induction of experimental diabetes

After four weeks, fasted animals (8 hours) received an intraperitoneal injection of STZ (Sigma-Aldrich, San Luis, MO, United States) diluted in sodium citrate buffer (0.01M and pH 4.5) at the doses of 30, 35 or 40 mg/kg (diabetic groups), or sodium citrate buffer (control group). Glycemia was measured after STZ injection (initial glycemia), and the animals were fed 6 hours later. Forty-eight hours after feeding, the animals fasted again for 8 hours, and the final glycemia was obtained using a portable glucometer (Injex Sens II). The animals were considered diabetic when glycemia values were ≥ 250 mg/dL^7^.

### Insulin tolerance test

Insulin tolerance test was determined four days before the end of the experiment. The test was performed in a fed state. The blood glucose was verified at time 0 (before intraperitoneal insulin administration) using a glucometer (Injex Sens II), through a small incision in the caudal vein. Afterwards, insulin (NovoRapid Penfill) was given intraperitoneally (1.5 IU insulin/kg of body weight), and blood glucose was measured at 5, 10, 12 and 15 minutes, using the adapted procedure of Ayoub *et al.* 14.

### Anthropometric evaluation

Weight gain, length, thoracic and abdominal circumference, and Lee’s index, which uses the body weight ratio related to length, were evaluated according to Novelli *et al.* 15.

### Euthanasia and sample collection

After fasting for 8 hours, the animals were anesthetized (inhaling) with isoflurane, and then blood collection was performed at the retro-orbital cavity. Death was confirmed by anesthesia overdose. Blood was centrifuged at 3,500 rpm/15 min to obtain serum. After euthanasia, the retroperitoneal, epididymal and perirenal fats of each animal were completely removed and weighed using an analytical balance for a later comparison among groups^16^. The adiposity index (AI) was calculated by the ratio (body fat/final body weight) × 100^17^. Liver, kidney and pancreas were also collected for histological evaluation.

### Biochemical evaluation

The following parameters were determined in the serum: total cholesterol, high-density lipoprotein (HDL), glucose, liver function markers (alanine aminotransferase – ALT, aspartate aminotransferase – AST, alanine aminotransferase), renal parameters (urea, creatinine, and uric acid), protein profile (albumin and total protein) and α-amylase using Cobas C111 of commercial kits (Roche, United States) and spectrophotometry.

### Histological analysis

Liver, kidney and pancreas were placed in 10% buffered formalin solution (for fixation) and then kept in 70% alcohol until paraffin embedding. After this, 5-μm thick sections were obtained. The slides were dewaxed and stained with hematoxylin and eosin. Microscopy (Leica Application Suite, version 4.0) was used for analysis.

The images were captured with × 40 magnification. Histological analyses were based on the parameters of:

- Pancreas: area and number of islets, cellular vacuolization of pancreatic islets, and the exocrine portion;
- Liver: hepatic steatosis and vasocongestion;
- Kidney: renal steatosis and renal tubule hydropic degeneration.

Pancreatic islet area analysis was performed using 20 digital images (TIFF 8-bit color, 3,264 × 2,448 pixels, × 200 magnification) for each pancreas (n = 6 /group). Nikon D3100 photo camera coupled with Zeiss Primo Star microscope was used. The program used to measure islet areas was Image Processing and Analysis in Java (ImageJ). Quantitative analysis of the number of pancreatic islets was performed using Leica DM500 microscope at ×50 magnification. Pancreatic fields were used (n = 6 animals/group). Methodology was based on the article by Gomes *et al.* 18.

### Statistical analysis

Data were analyzed by the software GraphPad Prism version 6.0 using analysis of variance (one-way ANOVA) by Tukey’s test. Values were considered significant when p < 0.05.

## Results

### Anthropometric parameters, water and food intake

Final weight, weight gain, Lee’s index, and water and food intake differed among groups (p < 0.05), as shown in Table 1.

**Table 1:** Anthropometric parameters, and water and food intake of control and diabetic groups (n=10)*.

	CONTROL	30STZ	35STZ	40STZ
	M±SEM	M±SEM	M±SEM	M±SEM
Initial body weight (g)	197.90±5.91	198.00±5.84	198.80±3.60	197.60±3.68
Final body weight (g)	410.60±9.73^a^	353.90±12.71^b^	327.60±14.28^b^	320.10±11.42^b^
Weight gain (g)	212.70±6.54^a^	155.90±9.00^b^	128.80±11.67^b^	122.50±10.01^b^
TC (cm)	18.03±0.54	18.28±0.25	17.62±0.40	17.89±0.34
AC (cm)	16.23±0.35	16.01±0.27	15.70±0.11	15.32±0.22
Lee’s index (g/cm^3^)	0.31±0.002^a^	0.30±0.001^b^	0.29±0.002^b^	0.30±0.002^b^
Chow intake (g/day)	26.45±0.62^a^	18.52±0.51^b^	20.53±0.75^bc^	22.85±0.49^c^
Water intake (mL/day)	39.96±1.30^b^	41.65±7.32^b^	71.34±7.83^a^	84.99±8.22^a^

* Different letters on the same line indicate significant difference by Tukey’s test (p <0.05); STZ: streptozotocin; M: mean; SEM: standard error of the mean; AC: abdominal circumference; TC: thoracic circumference.

### Glycemia before and after streptozotocin injection

The results of initial fasting glycemia and 48 hours after STZ injection are presented in Table 2.

**Table 2:** Fasting glycemia before and 48 hours after streptozotocin injection of control and diabetic groups (n = 10)*.

Glycemia (mg/dL)	CONTROL	30STZ	35STZ	40STZ
	M±SEM	M±SEM	M±SEM	M±SEM
Initial	144.20±3.94	154.60±1.65	151.80±3.73	148.00±2.10
48h after STZ	153.60±7.91^b^	178.50±7.36^b^	359.90±44.43^a^	454.60±40.86^a^

* Different letters on the same line indicate significant difference by Tukey’s test (p < 0.05); STZ: streptozotocin; M: mean; SEM: standard error ofthe mean.

### Weight of organs and tissues

Table 3 presents the weight of different organs and adipose tissue of the control and experimental groups. Only the values of the pancreas and adiposity index were different between groups (p < 0.05).

**Table 3:** Weight of organs and adipose tissue of control and diabetic groups (n = 10)*.

	CONTROL	30STZ	35STZ	40STZ
	M±SEM	M±SEM	M±SEM	M±SEM
Liver (g)	14.56±0.87	14.64±0.49	14.56±0.78	14.40±0.50
Kidney (g)	1.52±0.03	1.39±0.08	1.65±0.11	1.67±0.06
Pancreas (g)	1.61±0.07^a^	1.31±0.07^b^	1.23±0.07^b^	1.30±0.05^b^
**Adipose tissue**				
Epididymis (g)	3.95±0.35	4.74±0.52	4.32±0.50	3.91±0.47
Perirenal (g)	1.17±0.11	1.38±0.12	1.00±0.17	1.00±0.19
Retroperitoneal (g)	3.82±0.31	5.11±0.60	3.39±0.74	0.86±0.49
Adiposity index (%)	2.17±0.10^b^	3.17±0.14^a^	2.56±0.31^ab^	2.13±0.20^b^

* Different letters on the same line indicate significant difference by Tukey’s test (p < 0.05); STZ: streptozotocin; M: mean; SEM: standard error of the mean.

### Biochemical evaluation

Table 4 shows the biochemical parameters of the control and experimental groups. Glucose, HDL, total protein, urea, AST, ALT and α-amylase were different among groups.

**Table 4:** Biochemical parameters of control and diabetic groups (n = 10)*.

	CONTROL	30STZ	35STZ	40STZ
	M±SEM	M±SEM	M±SEM	M±SEM
Glucose (mg/dL)	98.83±2.00^c^	285.70±8.73^b^	310.80±6.37^a,b^	334.30±9.17^a^
Cholesterol (mg/dL)	51.77±3.45	58.78±4.18	47.57±1.94	48.69±1.67
HDL (mg/dL)	49.08±2.81^a^	50.02±3.65^a^	38.60±1.56^b^	38.88±1.14^b^
Total protein (g/L)	52.06±1.89^a^	51.22±2.52^a,b^	44.37±1.67^b^	45.24±1.01^a,b^
Creatinine (mg/dL)	0.43±0.03	0.57±0.06	0.49±0.03	0.057±0.05
Albumin (g/L)	42.97±2.15	42.69±2.40	36.19±1.39	38.66±1.29
Uric acid (mg/dL)	0.60±0.02	0.78±0.07	0.66±0.05	0.63±0.05
Urea (mg/dL)	25.34±1.30^c^	29.01±0.68^b,c^	30.34±1.15^b^	37.85±0.90^a^
AST (U/L)	41.97±2.06^c^	70.48±3.76^b^	85.71±2.31^a^	94.27±3.10^a^
ALT (U/L)	30.87±1.67^d^	62.29±2.34^c^	72.77±1.81^b^	82.14±2.88^a^
α-Amylase (U/L)	407.60±4.73^c^	589.00±77.90^b^	797.20±7.39^a^	887.70±8.03^a^

* Different letters on the same line indicate significant difference by Tukey’s test (p < 0.05); STZ: streptozotocin; M: mean; SEM: standard error of the mean; HDL: high-density lipoprotein; AST: aspartate aminotransferase; ALT: alanine aminotransferase.

### Insulin tolerance test

**Figure 2:**
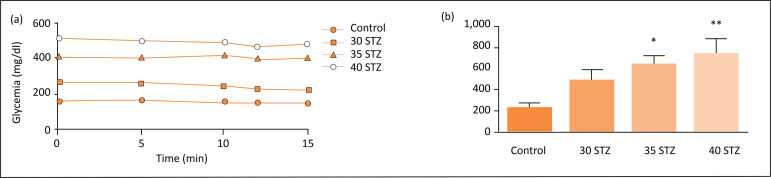
(**a**) Blood glucose levels in insulin sensitivity test applied at 0, 5, 10, 15 min; (**b**) area under the curve (AUC) of insulin sensitivity test.

Insulin tolerance test through time (Fig. 2a) and the area under the curve (AUC) (Fig. 2b) were higher in animals treated with 35 and 40 mg/kg STZ (p < 0.05).

*p <0.05 in relation to the control group; **p < 0.01 in relation to the control group. Values are presented as mean ± standard error of the mean (n=10); STZ: streptozotocin.

### Histological analysis of liver, pancreas, and kidney

**Figure 3:**
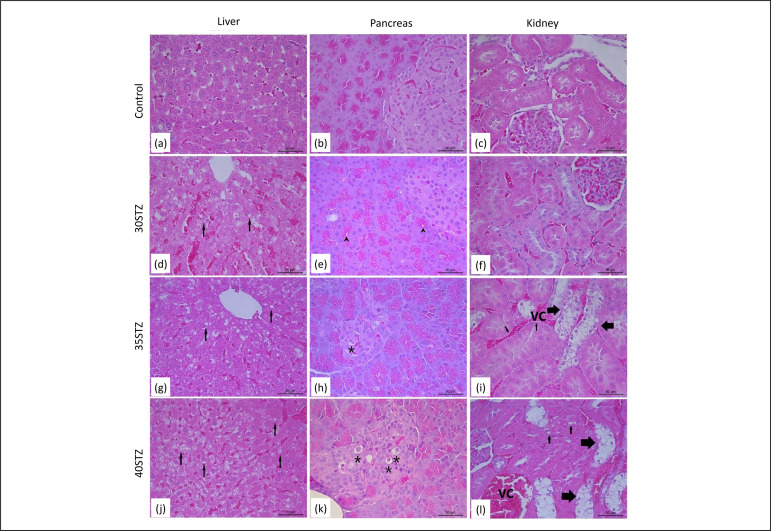
Effects of cafeteria diet and different doses of STZ on the histopathology of liver, pancreas, and kidney (H&E- Hematoxylin and eosin, ×40). (**a**) Control group represents liver with standard morphology; (**b**) Control group represents pancreas with standard morphology; (**b**) Control group represents kidney with standard morphology; (**d**) Group 30 mg/kg STZ: liver with mild steatosis (*thin arrow*); (**e**) Group 30 mg/kg STZ: pancreatic islets with reduced size and vacuolization (*arrowhead*) in pancreatic acini; (**f**) Group 30 mg/kg STZ represents kidney with standard morphology; (**g**) Group 35 mg/kg STZ: liver with mild steatosis (*thin arrow*); (**h**) Group 35 mg/kg STZ: small size pancreatic islets and apoptotic bodies (*asterisk*) on islet; (**i**) Group 35 mg/kg STZ: renal tubules with hydropic degeneration (*thick arrow*) and steatosis (*thin arrow*). Kidney with vasocongestion; (**j**) Group 40 mg/kg STZ: liver with moderate steatosis (*thin arrow*); (**k**) Group 40 mg/kg STZ: small-sized pancreatic islet and apoptotic bodies (*asterisk*) on the islet; (**l**) Group 40 mg/kg STZ: renal tubules with hydropic degeneration (*thick arrow*), steatosis (*thin arrow*). Kidney presents vasocongestion.

Histological analyses of liver, pancreas, and kidney are presented in Fig. 3. Animals treated with 35 and40 mg/kg STZ presented greater liver fat deposition (Fig. 3 g-j,respectively). The pancreas of the control group presented normal morphology (Fig. 3b), whereas animals in the STZ35 (Fig. 3h) and STZ40 (Fig. 3k) groups showed important changes in pancreatic morphology. The animals that received the doses of 35 and 40 mg/kg STZ presented renal damage (Fig. 3 i-l).

Based on the results presented in Table 5, we can see that the animals treated with 35 and 40 mg/kg STZ showed decrease in the area of pancreatic islets.

**Table 5:** Result of areas of pancreatic islets of control and diabetic groups (n = 10)&.

	CONTROL	30STZ	35STZ	40STZ
	M±SEM	M±SEM	M±SEM	M±SEM
Area islet (mm^2^)	28.80±2.26	30.51±2.87	15.01±1.23* #	21.03±1.71$ #
Number of islets	5.8±0.54	4.47±0.27	2.80±0.31* $	2.60±0.36* #

& The analysis of variance (ANOVA) test (one criterion) was used to check statistical difference, and Tukey’s test was used to locate the differences; STZ: streptozotocin; M: mean; SEM: standard error of the mean;

* control group compared to the other groups;

$ control group compared to the other groups;

# 30STZ group compared to the 35STZ and 40STZ groups).

## Discussion

Regarding final weight, the animals that received 30, 35 and 40 mg/kg STZ presented reduction of 13.8, 20.2 and 22% in weight in comparison to the control group, which is consistent with literature data^19^. Weight loss is a common symptom in untreated, decompensated diabetic^20^. Due to metabolic dysfunction, tissues cannot absorb glucose; the body creates pathways to obtain energy by increasing gluconeogenesis, lipolysis and ketone production, leading to weight loss^21^.

In the study by Campos *et al*.^22^, a correlation was observed between higher blood glucose values and lower weight in diabetic animals, indicating that the body’s inability to use glucose due to insulin resistance leads to a catabolic condition.

The lower weight gain observed in STZ-treated animals may also be related to a decreased food intake (Table 1). The higher food intake in animals fed with the control diet is corroborated by Hariri *et al.* 23. Animals fed with high-fat chow presented lower food intake when compared to animals fed with commercial chow. Rosado and Monteiro^24^ affirm that this finding can be explained by the release of the satiety cholecystokinin hormone after the intake of foods rich in lipids. Our findings also show that the lower weight gain in diabetic animals influenced Lee’s index as well. Lee’s index may reflect the degree of obesity^15^. However, this calculation does not evaluate the percentage of fat. Therefore, it is not possible to state that the animals in the control group are obese.

Animals in the 35 and 40 mg/kg STZ groups presented higher water intake compared to the control group. Our findings are supported by other studies^25^^,^ 26 and clinical symptoms found in human patients with DM2: polyuria and polydipsia^21^^,^ 27.

The use of low doses of STZ induces the death of pancreatic beta cells through the alkylation of DNA, leading to a slight impairment of insulin secretion with subsequent hyperglycemia, whereas hyperlipidic diets lead to obesity, insulin resistance and mild hyperglycemia^28^^,^ 29. In the present study, animals treated with 35 and40 mg/kg of STZ presented a glycemia higher than250 mg/dL, demonstrating greater efficiency in the induction of experimental DM2, in accordance with Castro *et al*.^11^. Furthermore, the reduction of islet size was also found by Castro *et al*.^11^, who evaluated animals treated with35 mg/kg STZ receiving high-fat diet containing standard chow, peanut, milk chocolate and sweet biscuits at a ratio of 3:2:2:1. Thus, the combination of a low dose of STZ and hyperlipidic diets has shown to be an effective model to simulate the metabolic characteristics of DM2 in humans, like insulin resistance^9^^,^ 11.

STZ-treated groups presented decreased pancreatic mass compared to the control group. This result is similar to the ones obtained by Castro *et al*.^11^, who observed decrease in the pancreas weight of diabetic animals. This result can be explained by the action of STZ in destroying beta cells of the islets of Langerhans, damaging cellular functionality and, consequently, decreasing the production and insulin release, as well as reducing the size of the islets^11^^,^ 30.

The group treated with 30 mg/kg STZ presented a higher AI compared to the control group. Leopoldo*et al*.^31^ conducted a study comparing obese animals fed with a high-fat diet with a control group (animals fed with a standard diet) and found similar results.

Animals treated with STZ presented higher levels of blood glucose compared to the control group. Increased blood glucose is a common characteristic of uncontrolled diabetes^21^. Magalhães *et al*.^32^ performed a similar study with a high-fat diet and animals from another bioterium and found that only animals that received the dose of40 mg/kg presented blood glucose higher than the control animals. In our findings, all STZ doses (30, 35 and 40 mg/kg) presented higher glycemia than the control group, above the reference value for diabetic animals (250 mg/dL)^11^.

In DM2, triglyceride levels increase and HDL ones decrease^32^^,^ 33. Animals treated with 35 and 40 mg/kg STZ presented low HDL cholesterol values. Jiao *et al*.^33^ found similar results. This can be attributed to an excess of circulating fatty acids derived from adipose tissue in the liver. With a higher production of fatty acids, there is decrease in insulin sensitivity in muscle tissues^34^. Adiels *et al*.^35^ stated that DM2 and insulin resistance are associated with excess of liver production of very-low-density lipoprotein (VLDL).

A decrease in total proteins was observed in animals that received 35 mg/kg STZ in comparison to the control group, which may indicate hepatic or renal disorder or decompensated diabetes. According to Castaneda *et al*.^36^, decompensated DM2 is associated with altered protein profiles in the body, since insulin resistance (as observed in the present study, Fig. 2) may lead to muscle mass loss, which is indicated by the decrease in serum proteins.

The groups treated with 35 and 40 mg/kg STZ presented increased values of urea compared to the control group. High urea levels might suggest renal dysfunction^37^. Uncontrolled diabetes leads to serious renal damage over time, being one of the main causes of renal failure^3^. Thus, in our study, the association of low doses of STZ with a cafeteria diet was efficient in simulating deleterious effects on the animals’ kidneys, corroborating the observed effects of diabetes in humans.

Animals that received STZ presented higher levels of AST and ALT compared to the control group. In the study by Ulla *et al*.^38^, rats fed with high-fat diet displayed high ALT and AST activity in plasma, as well as a fatty liver, compatible with non-alcoholic liver disease associated with high-fat diets and liver oxidative stress. The occurrence of non-alcoholic liver disease is considerably higher in diabetics compared to non-diabetics^39^.

The α-amylase values in the 35 and 40 mg/kg STZ groups were higher than in the control group (Table 4). A major product of the secretion of pancreas and salivary glands, α-amylase is considered the key enzyme in the digestive system, a prerequisite for the onset of the digestion process^40^. Starch digestion occurs mainly in the small intestine by the action of pancreatic enzymes such as α-amylase, which degrades carbohydrates into oligosaccharides and disaccharides, and by α-glycosidases in the membrane of intestinal cells, which hydrolyze oligo- and disaccharides into monosaccharides^40^. Degradation of dietary starch advances fast and leads to high postprandial hyperglycemia. Amylase is also widely used as a serum marker of pancreatic inflammation^40^.

The faster blood glucose decreases after insulin administration; a greater insulin sensitivity occurs^41^. In our study, there was increase in serum glucose levels in the groups receiving STZ, as expected (Fig. 2a). By AUC analysis (Fig. 2b), the animals in the groups receiving 35 and 40 mg/kg STZ showed larger AUC, indicating higher insulin resistance compared to the control group. Castro *et al*.^11^ also observed an increased insulin resistance in diabetic Wistar rats consuming a high-fat diet at the dose of 35 mg/kg STZ.

The animals that received the dose of 30 mg/kg STZ did not show insulin resistance (Fig. 2b), indicating that the dose of STZ used did not cause sufficient damage to the pancreatic beta cells, to the point of causing insulin resistance in the animals in this group, as observed in groups 35STZ and 40STZ.

According to Srinivasan *et al*.^29^ and Wilson and Islam^42^, an experimental model that combines high-fat diet and low doses of STZ is effective in causing insulin resistance, as well as being efficient in simulating the effects of DM2. Our findings evidence that, the higher the concentration of STZ, the higher the insulin resistance. Thus, 35 and 40 mg/kg STZ associated with a cafeteria-type diet were more efficient in causing insulin resistance. Thirty mg/kg of STZ produced significant hyperglycemia (Table 4), but did not cause significant insulin resistance in the animal model studied (Fig. 2b).

The insulin resistance and DM2 associated or not with metabolic syndrome lead to the release of fatty acids by adipocytes, which accumulate in the liver tissue favoring lipogenesis and increasing liver triglycerides^43^^-^ 45. It represents an important risk factor for the development of hepatic steatosis. The 35 and 40 mg/kg STZ groups presented greater insulin resistance (Fig. 2), confirming that DM2 is a major contributor to hepatic steatosis^43^^-^ 45. The results obtained in the present study are also similar to the data by Buettner *et al*.^46^, who observed hepatic steatosis in animals receiving a high-fat diet containing lard.

The 35 (Fig. 3h) and 40 mg/kg STZ (Fig. 3k) groups showed decrease in pancreatic islets and apoptotic bodies in the islet, corroborating with the data observed in Tables 2 and 5, in which animals treated with 35 and 40 mg/kg STZ presented a lighter pancreas, with decreased area and number of pancreatic islets. These data confirm the diabetogenic action of STZ associated with a cafeteria diet, which leads to irreversible damage to the pancreatic beta cells and to a loss of cellular function to produce and release insulin^11^.

Decompensated diabetes can lead to kidney failure, and approximately 10% of deaths in people with DM2 are attributed to renal failure^3^. In the present study, the histopathological evaluation of the kidney showed that animals of the control and 30 mg/kg STZ groups presented standard morphology (Fig. 3f), whereas animals that received the doses of 35 and 40 mg/kg STZ presented renal damage (Fig. 3 i-l). Our findings also show (Table 4) that animals in 35 and 40 mg/kg STZ groups had high urea levels, indicating impaired renal function in animals^47^.

## Conclusions

Our study proposes an experimental model for the development of DM2 in Wistar rats using a cafeteria diet and low doses of STZ. Our model proved to be an efficient, low-cost model without loss of animals during the experiment. The doses of 35 and 40 mg/kg STZ were effective in simulating metabolic characteristics of DM2 in humans. It was confirmed by the presence of polydipsia, hyperglycemia, altered biochemical tests, insulin resistance and damages to the liver, pancreas, and kidney. Thus, due to economic aspects and the high success rate, we recommend using 35 mg/kg STZ for diabetes induction associated with a cafeteria diet. Our findings are useful for future studies involving DM2 pathogenesis, environmental factor interactions, and evaluation of new treatments.
